# Proposed Mechanistic Axis of Infections and mTOR Hyperactivation: A Multidisciplinary Review of Immune, Rheumatologic, and Psychiatric Links

**DOI:** 10.3390/children12121603

**Published:** 2025-11-25

**Authors:** Giovanni Fronticelli Baldelli, Danilo Buonsenso

**Affiliations:** 1 Medicine and Surgery, Catholic University of Rome, 00168 Roma, Italy; 2 Department of Woman and Child Health and Public Health, Fondazione Policlinico Universitario A. Gemelli IRCCS, 00168 Rome, Italy; 3 Centro di Salute Globale, Università Cattolica del Sacro Cuore, 00168 Roma, Italy; 4 Area Pediatrica, Dipartimento di Scienze Della Vita e di Sanità Pubblica, Università Cattolica del Sacro Cuore, 00168 Roma, Italy

**Keywords:** infections, mTOR, neuroinflammation, psychiatry

## Abstract

Early-life infections can produce durable changes in immune function and behavior. We propose a mechanistic hypothesis positioning the mechanistic target of rapamycin (mTOR) as the link between peripheral inflammation and central nervous system dysfunction in pediatric post-infectious syndromes. Based on clinical, translational, and experimental literature, we outline a stepwise pathway. First, sustained mTOR activation skews T-cell and macrophage differentiation toward pro-inflammatory and autoimmune states. Second, endothelial mTOR signaling weakens tight junctions and increases vesicular transport, compromising blood–brain barrier integrity. Third, cytokines and sometimes autoreactive cells enter the brain and engage mTOR in microglia and neurons, driving neuroinflammation, impaired synaptic maintenance and plasticity, and neurotransmitter disruption. This framework accounts for features observed in Long COVID, myalgic encephalomyelitis/chronic fatigue syndrome (ME/CFS), and pediatric acute neuropsychiatry syndrome (PANS/PANDAS) and yields testable predictions on pathway activity and barrier permeability. It also motivates targeted interventions that modulate mTOR-related processes in immune and endothelial compartments and within neural circuits in children. So, this article aims to outline a mechanistic framework linking infection-driven mTOR activation to post-infectious neuropsychiatric syndromes.

## 1. Introduction

Reports since the start of the COVID-19 pandemic describe increases in chronic fatigue syndromes, neurocognitive difficulties, and prolonged school absenteeism in children, raising concerns about long-term neuroimmune effects after infection [1]. These observations refocus attention on pathways that couple post-infectious immune activation to persistent, multisystem disturbances.

mTOR sits at a convergence point of immune and neural signaling. In the periphery, sustained mTOR activation reshapes T-cell and macrophage differentiation and can favor pro-inflammatory, autoimmune states. At the brain endothelium, mTOR influences tight-junction proteins and vesicular transport, with consequences for blood–brain barrier (BBB) integrity and the trafficking of cytokines and, at times, autoreactive cells into the central nervous system (CNS). Within the CNS, mTOR signaling in microglia and neurons regulates neuroinflammation, synaptic maintenance, plasticity, and neurotransmission, a process relevant to anxiety, mood, and psychotic symptoms.

**Figure 1:**
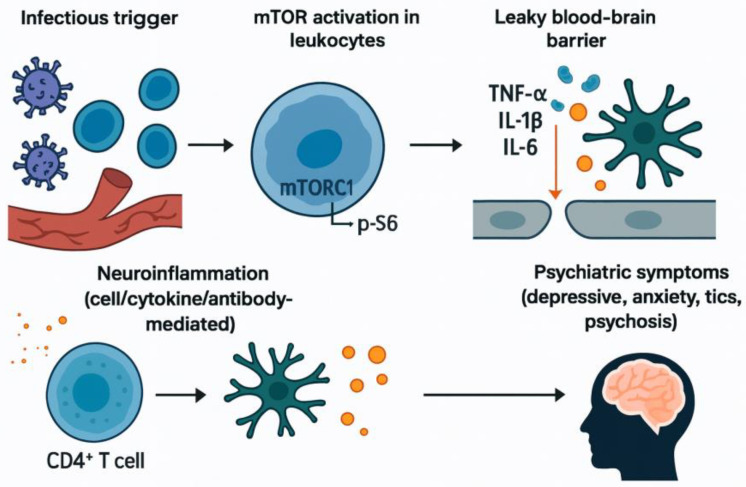
Proposed pathway linking infection, mTOR activation, and psychiatric symptoms. Infectious stimuli activate mTORC1 signaling in leukocytes, marked by phosphorylation of S6. This activation promotes cytokine release (TNF-α, IL-1β, IL-6), increasing blood–brain barrier permeability and driving neuroinflammation through CD4^+^ T-cell and antibody-mediated mechanisms. The resulting immune dysregulation is hypothesized to contribute to psychiatric manifestations including depression, anxiety, tics, and psychosis.

We hypothesize that infection-driven perturbations of mTOR form a mechanistic axis linking peripheral immune dysregulation, BBB compromise, and neural circuit dysfunction (Figure 1). This article synthesizes clinical and experimental evidence across entities such as Long COVID, ME/CFS, and PANS/PANDAS, and outlines testable predictions and therapeutic implications.

## 2. The Case

A previously healthy 14-year-old girl developed persistent symptoms following mild COVID-19 in October 2020. One month after infection, she experienced ongoing headache, chest pain, fatigue, and sinus tachycardia, leading to multiple emergency visits. Extensive cardiopulmonary investigations were normal except for poor exercise tolerance and early tachycardia on exertion. Seven months post-infection, symptoms persisted, with a 6 min walk test showing marked tachycardia, up to 155 bpm, and easy fatigability. Immunological assessment revealed elevated IL-6, IL-1, and TNFα, an increased effector T-cell/regulatory T-cell ratio, and reduced CD27+ memory B lymphocytes, indicating a sustained pro-inflammatory state. Anti-SARS-CoV-2 IgA and IgG levels remained high. This case highlights chronic symptomatology and ongoing immune activation months after acute COVID-19 in an adolescent without prior comorbidities. As this case would still be considered idiopathic and without a possible therapy, we explore how infections can trigger such subtle inflammatory responses and how this can be tackled with specific studies.

## 3. Interactions Between Infection and mTOR Pathways

Some infections do not end with symptom resolution; latent pathogens like HSV and EBV can drive persistent immune activation [2]. This chronic, post-infectious inflammation can rewire the physiological role of a series of molecular agents, such as the mammalian target of rapamycin (mTOR), a serine/threonine kinase that regulates cell growth, apoptosis, autophagy and proliferation [3]. Direct human evidence of mTOR dysregulation in Lyme disease is lacking; however, Borrelia modulates immunometabolic programs, including PI3K/Akt/mTOR, in human immune cells ex vivo, and Borrelia-induced cytokine production depends on glycolysis/HIF-1α. These observations make mTOR involvement plausible on mechanistic grounds, but not proven in patients [4,5,6,7].

Herpesviruses (HSV-1, VZV, EBV) and coronaviruses modulate host PI3K–Akt–mTOR–autophagy pathways to support replication, persistence and immune evasion. Autophagy, while generally antiviral, can be repurposed by viruses [8]. For example, the PRV US3 protein inhibits autophagy through Akt/mTOR activation [9]. EBV latent protein LMP1 activates both mTORC1 and mTORC2, and inhibiting mTORC2 reduces EBV production [10]. More broadly, viruses exploit the mTOR axis at multiple nodes to benefit their life cycle [11]. In SARS-CoV-2, infected airway/ALI cultures and human autopsy lungs show increased readouts of mTORC1 activity and broad metabolic rewiring; endothelial dysfunction is a consistent feature of COVID-19, but direct in vivo evidence that sustained mTORC1 activation in human monocytes or endothelium drives post-acute inflammation remains limited and mostly inferential [12,13,14]. HSV-1 interfaces with PI3K–AKT–mTOR and autophagy: viral proteins (e.g., ICP34.5, Us3) counter autophagy, while neuronal mTORC2 supports cell survival during infection, patterns linked to latency/reactivation dynamics rather than a single directional effect [15,16]. A serious sequela of HSV encephalitis is autoimmune encephalitis, driven by persistent CNS inflammation even after the virus is cleared; milder or differently timed inflammatory responses may instead manifest as anxiety or depressive symptoms rather than acute encephalitis.

## 4. Pathogenic Autoimmune Shift

Multiple studies have established mTOR as a central molecular switch governing the fate of T cells: mTOR is a central node in CD4^+^ T-cell fate. mTORC1 is required for Th1 and Th17, while mTORC2 supports Th2; broad mTOR loss favors FoxP3^+^ Treg induction [17,18,19]. p70S6K1 downstream of mTORC1 is specifically needed for Th17 [20]. Reduced Treg number/function undermines peripheral tolerance [21,22]. This pro-inflammatory skew is reinforced by mTORC1-driven glycolysis and HIF-1α, which promote Th17 and antagonize Treg [23,24]. mTORC1 and mTORC2 also support Tfh differentiation [25].

In CD8^+^ T cells, mTOR signaling shapes effector–memory fate. Elevated mTORC1 activity drives terminal effector differentiation with high cytotoxic function and IFN-γ, whereas dampening mTORC1 favors long-lived memory; genetic dissection is associated with mTORC1 and mTORC2 serving distinct contributions to effector and memory programming [26,27]. Sustained effector-biased states during chronic viral stimulation can align with inflammation and features of exhaustion, though direct human causal links are limited. In macrophages, mTORC1 activity promotes M1-like pro-inflammatory programs and restrains M2 polarization, while mTORC2supports type-2/M2 programs; these effects are context- and timing-dependent [28,29,30,31].

## 5. mTOR-Mediated Endothelial Barrier Breakdown

In brain endothelium, mTOR helps maintain blood–brain barrier (BBB) homeostasis. In preclinical models, excessive mTOR activity is associated with barrier leakage, whereas mTOR inhibition preserves tight-junction integrity and reduces permeability [32,33]. Tight-junction components such as claudin-5 and occludin are critical for paracellular sealing [34]; hyperactivation contexts often show their loss, and rapamycin can preserve these proteins in injury models [33]. Transcellular flux is also relevant: the MFSD2A pathway restrains endothelial vesicle-mediated transcytosis, and a brain endothelial PTEN–AKT–NEDD4-2–MFSD2A axis modulates this process; perturbation increases vesicle trafficking and barrier permeability [35,36,37]. While AKT can feed into mTOR signaling, direct human evidence that mTORC1 alone drives increased BBB transcytosis is limited; most support remains preclinical. These paracellular and transcellular changes can expose the CNS to peripheral cytokines and immune cells and are considered early features of neuroinflammation in several conditions. Developmentally, BBB function is established early yet continues to mature postnatally, and sex hormones modulate cerebrovascular and BBB phenotypes across the lifespan, factors that may shape age- and sex-dependent vulnerability to mTOR-linked barrier dysfunction and psychiatric risk [38,39,40].

## 6. CNS mTOR Dysregulation, Neuroinflammation and Psychiatric Outcomes

Peripheral inflammatory mediators such as cytokines can penetrate a compromised blood–brain barrier and influence central nervous system immunity. In glial cells, activation of mTOR signaling has been linked to a shift toward pro-inflammatory microglial and astrocytic phenotypes. Such changes can impair neuronal homeostasis by disturbing synaptic support and plasticity [41].

In the CNS, mTOR regulates both neuronal and glial function. Its hyperactivation contributes to excessive cytokine signaling and impaired autophagy in glial cells, fueling neuroinflammatory cascades, while in neurons it disrupts synaptic plasticity and leads to cognitive and behavioral deficits: in mouse models of Alzheimer’s disease, hyperactivation of the mTOR pathway impairs synaptic maintenance and is linked to memory deficits [42,43]. Also, in models of Alzheimer’s and vascular cognitive impairment, rapamycin mitigates junctional loss and preserves endothelial barrier function [33].

Although the downstream mechanisms underlying these dysfunctions in Alzheimer’s models remain unclear, and it is uncertain whether they overlap with the post-infectious processes proposed here, these findings nonetheless strengthen the evidence implicating mTOR in the development of neuropsychiatric symptoms [44].

Aberrant mTOR activity is a recurring feature in psychiatric disorders such as schizophrenia and bipolar disorder, where it contributes to disrupted synaptic pruning, excitatory–inhibitory imbalance, and altered glutamatergic signaling within cortical microcircuits [45,46,47].

mTOR dysregulation influences excitatory synaptic function by altering AMPA/NMDA receptor dynamics, dendritic spine structure, and local protein synthesis. In models of stress-related depression, reduced BDNF–mTOR signaling in the hippocampus is associated with synaptic atrophy and depressive-like behaviors, while enhancement of this pathway by agents such as S-ketamine restores plasticity and improves mood [48,49]. Thus, whether through hyperactivation or suppression, mTOR imbalance disrupts synaptic integrity, compromises neuronal resilience, and underpins a spectrum of psychiatric symptoms.

## 7. Clinical Correlates

Many medical and psychiatric conditions remain categorized as “idiopathic,” reflecting the current inability to trace their pathogenesis to a single known trigger. Increasingly, chronic inflammation and subtle immune dysregulation are being recognized as shared features in a wide range of these disorders. Notably, post-infectious syndromes such as PANS/PANDAS and Long COVID, once considered idiopathic, have demonstrated clear links between peripheral immune activation and cognitive and behavioral symptoms, including mood lability, anxiety, obsessive–compulsive tendencies, and cognitive dysfunction, as discussed in more detail later. These overlaps underscore the role of inflammatory mediators in shaping neural function, particularly when immune signaling crosses into the CNS through compromised barriers. This growing body of evidence challenges the traditional dichotomy between “organic” and “functional” illnesses, pointing instead toward a unified framework in which neuroimmune mechanisms may underlie many conditions historically labeled as idiopathic.

### 7.1. PANS/PANDAS

Among the most discussed, we certainly find PANS/PANDAS: this condition is thought to follow streptococcal or other infections, with an increased frequency of Th17 lymphocytes, which are thought to contribute to disease pathogenesis by promoting neuroinflammatory responses [50]. Elevated levels of IL-17 and IFN-γ promote chronic systemic inflammation, a hallmark of PANS/PANDAS [51]. Based on preclinical data, we hypothesize that mTOR-mediated changes in vascular endothelium impair tight junctions and upregulate adhesion molecules, facilitating the passage of cytokines, and in acute cases, autoantibodies, into the CNS. Microglial neuroinflammation in basal ganglia circuits has been demonstrated in PANDAS by TSPO-PET. Independently, mTORC1-driven translation amplifies microglial priming in vivo. Together, these findings support the hypothesis that dysregulated microglial mTOR could sustain basal-ganglia inflammation contributing to sudden-onset OCD, tics, and anxiety [52,53]. Reports examining long-term pediatric infectious outcomes have highlighted glial and vascular involvement in post-infectious neuropsychiatric syndromes, reinforcing the plausibility of mTOR-sensitive pathways as drivers of enduring CNS dysfunction [1].

### 7.2. Long COVID

Acute SARS-CoV-2 infection engages host PI3K/AKT/mTOR signaling in endothelial and epithelial cells, aiding viral replication and immune evasion; similar pathway activation in immune cells has been observed, though monocyte-specific mTOR hijacking is less well established [54,55]. Even after SARS-CoV-2 is cleared from circulation, evidence supports the hypothesis that systemic inflammation can persist. Sustained activation of pro-inflammatory pathways, including mTOR-related signaling, may drive ongoing production of cytokines such as IL-1β and IL-17 in immune and endothelial cells, contributing to the chronic low-grade inflammation characteristic of Long COVID [56]. Persistent inflammatory signaling in Long COVID contributes to mTORC1 hyperactivation in endothelial cells, thus possibly impairing tight junction integrity and sustaining BBB leakiness. This dysfunction enables immune signals and inflammatory mediators to reach the CNS long after the acute infection resolves, supporting a model of chronic neuroimmune activation. Within the brain, aberrant mTOR signaling in microglia and neurons is thought to underlie persistent symptoms such as cognitive dysfunction, fatigue, dysautonomia, mood disturbances, hallmarks of Long COVID and in children. Recent integrative analyses of immune and neurological biomarkers suggest that these sequelae emerge from persistent mTOR and MAPK dysregulation across organ systems, supporting a model of multisystem, post-viral neuroimmune reprogramming [57]. The most affected systems appear to be the cardiopulmonary system, the CNS (as discussed), and some evidence points to disturbances in the gastrointestinal tract [58,59,60].

### 7.3. ME/CFS

The last condition we discuss is Myalgic Encephalomyelitis/Chronic Fatigue Syndrome (ME/CFS), a disabling condition often following infections such as EBV and human herpesvirus-6 (HHV-6), both implicated in chronic immune activation and viral reactivation in a subset of patients. While direct evidence of mTORC1 hyperactivity in immune cells is limited, studies have consistently shown altered immunometabolism and pro-inflammatory cytokine profiles, particularly elevated IL-6 and TNF-α [61], which suggest possible involvement of mTOR-regulated pathways in monocytes and T cells. These changes are accompanied by immune exhaustion and impaired memory T-cell generation, contributing to a persistent inflammatory state [62]. Functional and structural neuroimaging studies show abnormalities in the brainstem and cortical regions, including reduced cerebral blood flow, altered brainstem connectivity, and changes in glial activity, findings correlated with cognitive symptoms such as post-exertional malaise, sleep disruption, and “brain fog” [63]. mTOR has also been associated with mitochondrial health and function, contributing to normal mitophagy process and turnover [64]: dysfunction of this organelle has been implicated in fatigue syndromes (as primary mitochondrial diseases display severe muscle fatigue among other clinical symptoms) [65] and neuropsychiatric disorders [66]. Despite increasing biological insights into these pathways, ME/CFS remains underrecognized in many clinical settings, and its pathophysiology continues to be debated, particularly in the context of its complex symptomatology and overlapping features with other post-viral syndromes [67].

Although no peer-reviewed interventional trials have reported outcomes for rapamycin/sirolimus in PANDAS, Long COVID, or ME/CFS, a pre-print study has linked low-dose rapamycin (6 mg/week) with a strong recovery in about 70% of patients [68], and there is an ongoing clinical trial with the same treatment with a once weekly oral rapamycin dose. The available evidence is summarized in Table 1.

## 8. Limitations

The immune system operates through numerous interconnected molecular switches and signaling pathways, making it implausible for a single mediator to act as the sole driver. Other cascades, such as NF-κB and JAK/STAT, also contribute to cytokine release and blood–brain barrier disruption, so mTOR is unlikely to serve as the exclusive central mechanism. While mTOR may influence maladaptive immune responses, the extensive crosstalk among inflammatory pathways makes attributing all dysfunction to one signaling route an oversimplification.

Moreover, human evidence linking mTOR hyperactivity to neuropsychiatric symptoms remains limited: outside monogenic mTORopathies it is largely observational or ex vivo [69]. Where causal tests exist, randomized, placebo-controlled everolimus trials in tuberous sclerosis complex and PTEN hamartoma tumor syndrome did not improve prespecified neurocognitive/behavioral composites over 6 months [70,71]. In depression, rapamycin pretreatment before ketamine failed to blunt the acute antidepressant response and may have prolonged benefit, indicating pathway modulation without establishing mTOR hyperactivity as a primary causal driver [72].

Direct human evidence that endothelial mTOR activation increases blood–brain barrier permeability remains limited. In vivo human imaging suggests BBB leakage in conditions such as small vessel disease, but these studies do not directly point to mTOR [73]. In cerebral cavernous malformations, endothelial mTORC1 activation has been observed, though its relationship to barrier dysfunction is only correlative [74]. By contrast, rodent and human-cell BBB models show that pharmacological mTOR inhibition preserves tight-junction proteins and reduces paracellular leak [32,34].

## 9. Conclusions and Future Directions

The unexpected increase in “idiopathic” conditions at the beginning of the COVID-19 pandemic opened new possibilities for understanding how infections can trigger complex, chronic, multisystemic diseases that still lack a known and defined pathogenesis. Indeed, SARS-CoV-2 has been a unique model not only for the attention it received but also because, for several months, it was one of the few viruses, if not the only virus, circulating, and, therefore, it was easier to link the beginning of a cluster of symptoms with a specific viral infection. The observations from COVID-19 and Long COVID are leading to the development of new models of chronic conditions (including mental illnesses and chronic fatigue syndromes) that have moved beyond the sole transmitter imbalances to include systemic and neuroinflammation and disrupted synaptic plasticity. Within this framework, mTOR would hypothetically be viewed as an integrator, linking immune-cell programs to neuronal structural and functional plasticity.

Much of this evidence is still emerging, so a priority is to test, in defined endotypes, whether mTOR dysregulation contributes to inflammation-driven conditions and whether its modulation shifts mechanistic and clinical endpoints. Addressing this will require redesigned, developmentally informed studies beginning in childhood to map how immune maturation and dysregulation relate to new-onset autoimmune-linked psychiatric presentations [75]. Importantly, having pediatric populations included in such a study (both preclinical and clinical) should be a priority, as pediatric patients usually do not have the same number of comorbidities as adults, which may interfere with the interpretation of results of basic science studies but also with responses to therapeutics. Importantly, these observations should facilitate the inclusion of pediatric patients in mTOR inhibitor trials, as adult trials in the field have already started.

## Figures and Tables

**Table 1:** Available evidence on the role of mTor in post-infectious syndromes.

Condition	mTOR Mechanism	Evidence Type	Reference
ME/CFS	Altered immunometabolism and cytokine signaling suggest mTOR involvement in monocytes and T cells; neural hypoperfusion and glial activity changes may reflect downstream mTOR-related effects.	Observational clinical and neuroimaging studies	[61,62,63,64,65,66,67]
Long COVID	Persistent mTORC1 activation in immune and endothelial cells may maintain systemic inflammation and BBB leakiness; microglial and neuronal dysregulation could explain chronic cognitive, affective, and autonomic symptoms.	Human tissue, autopsy, and biomarker studies	[54,55,56,57,58,59,60]
PANS/PANDAS	mTOR-mediated endothelial dysfunction may impair tight junctions and upregulate adhesion molecules, allowing cytokines and autoantibodies to enter the CNS; microglial mTORC1 activation may sustain basal-ganglia inflammation linked to sudden-onset OCD, tics, and anxiety.	Preclinical and imaging evidence; inferential human data	[50,51,52,53]

## Data Availability

No new data were created or analyzed in this study.
